# Ecological Networks in Stored Grain: Key Postharvest Nodes for Emerging Pests, Pathogens, and Mycotoxins

**DOI:** 10.1093/biosci/biv122

**Published:** 2015-09-09

**Authors:** John F. Hernandez Nopsa, Gregory J. Daglish, David W. Hagstrum, John F. Leslie, Thomas W. Phillips, Caterina Scoglio, Sara Thomas-Sharma, Gimme H. Walter, Karen A. Garrett

**Affiliations:** John F. Hernandez Nopsa (nopsa@ufl.edu) is a postdoctoral research associate in the Institute for Sustainable Food Systems and the Plant Pathology Department at the University of Florida (UF), in Gainesville, and was formerly a postdoctoral research associate in the Department of Plant Pathology at Kansas State University (KSU), in Manhattan, and affiliated with the Plant Biosecurity Cooperative Research Centre (CRC), in Canberra, Australia. Gregory J. Daglish is a principal research scientist at the Department of Agriculture and Fisheries, in Queensland, and is affiliated with the CRC. David W. Hagstrum is a professor in the Department of Entomology at KSU. John F. Leslie is a university distinguished professor in the Department of Plant Pathology at KSU and is affiliated with the CRC. Thomas W. Phillips is Professor Donald A. Wilbur, Sr. Endowed Professor in Stored-Product Protection in the Department of Entomology at KSU and is affiliated with the CRC. Caterina Scoglio is a professor in the Department of Electrical and Computer Engineering at KSU and is affiliated with the CRC. Sara Thomas-Sharma was a postdoctoral research associate in the Department of Plant Pathology at KSU and is currently in the Department of Plant Pathology at the University of Wisconsin–Madison. Gimme H. Walter is a professor in the School of Biological Sciences at the University of Queensland and is affiliated with the CRC. Karen A. Garrett (karengarrett@ufl.edu) is a preeminent professor in the Institute for Sustainable Food Systems and Plant Pathology Department at UF, is affiliated with the CRC, and was formerly a professor in the Department of Plant Pathology at KSU.

**Keywords:** insect pests, mycotoxins, postharvest networks, stored grain, wheat transportation

## Abstract

Wheat is at peak quality soon after harvest. Subsequently, diverse biota use wheat as a resource in storage, including insects and mycotoxin-producing fungi. Transportation networks for stored grain are crucial to food security and provide a model system for an analysis of the population structure, evolution, and dispersal of biota in networks. We evaluated the structure of rail networks for grain transport in the United States and Eastern Australia to identify the shortest paths for the anthropogenic dispersal of pests and mycotoxins, as well as the major sources, sinks, and bridges for movement. We found important differences in the risk profile in these two countries and identified priority control points for sampling, detection, and management. An understanding of these key locations and roles within the network is a new type of basic research result in postharvest science and will provide insights for the integrated pest management of high-risk subpopulations, such as pesticide-resistant insect pests.

**Stored grain provides a unique sheltered environment** with abundant and nutritious food for suitably adapted species. It also provides the potential for the local and global dispersal of these species through grain transportation and marketing networks. The links connecting stored-grain facilities extend from the farm across the globe as grain moves through transportation systems, including trucking, rail, barges, and ocean-going ships. Grain in the United States is typically stored for 6 months or longer (Hagstrum et al. 1999), long enough to be affected by pest infestations (where we use *pest* to refer to insects and mites that reduce grain quality or quantity), rodents, fungal growth, and additional mycotoxin contamination (Flinn et al. 2003, 2007, 2010). Failure to control these problems when they initially occur in storage (or in the field) can result in the movement of the problem through the system (Flinn et al. 2003), with the potential for the extensive contamination and destruction of stored grain, as well as devastating economic losses and threats to food security. Mycotoxins in grain, produced by fungi in the field or during storage, are serious threats to food safety and are hazardous to humans and livestock. Losses to stored-grain pests ranged from 9% in the United States to 20% in developing countries (Pimentel 1991). The estimated annual stored-grain loss to mycotoxin contamination in the United States alone was $932 million (Richard et al. 2003).

Wheat is the second highest total value food staple and the fourth highest volume crop globally (FAO 2013), providing about 55% of the carbohydrates and 20% of the calories consumed by humans. Our analyses focus on wheat in the United States and Queensland (Qld), Eastern Australia. The United States was the fourth largest wheat producer worldwide in 2011 (FAO 2013). A total of 62 million tons was produced in 2012, and five states accounted for more than half of this production: Kansas, North Dakota, Montana, Oklahoma, and Washington (NASS 2013). Australia was the sixth largest producer of wheat in 2011, at 27.5 million tons (FAO 2013). In the United States and Australia, the nodes in networks of stored wheat are primarily locations (states and towns) that contain commercial facilities (elevators, silos, or silo bags; figure 1; Flinn et al. 2007, Ridley et al. 2011a).

**Figure 1.**
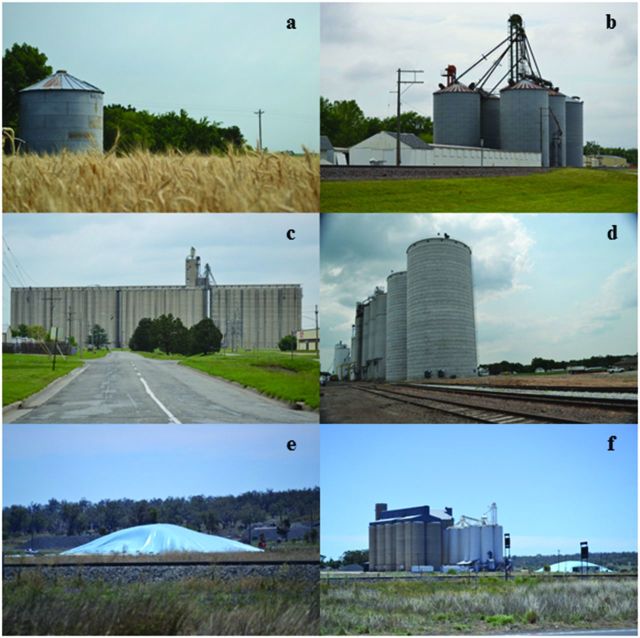
Grain-storage structures in Kansas (KS), United States, and Queensland (Qld), Australia. (a) A farm bin near Waterville, KS. (b) A country elevator in Rossville, KS. (c) A terminal elevator in Topeka, KS. (d) A rail-loading elevator in Hanover, KS. (e) A silo bag in Qld. (f) A concrete silo next to a depot near Toowoomba, Qld. Photographs: John F. Hernandez Nopsa.

Understanding the structure and dynamics of stored-grain networks will give new insight not only into the movement of grain but also into which nodes (e.g., silos, states) are located on the shortest and most likely paths for the spread of mycotoxins or pests. This information provides an important foundation for sampling and developing strategies in integrated pest management (IPM). IPM offers an alternative to the common traditional pest-control practices of routine fumigation not based on need (Reed et al. 2003, Flinn et al. 2007). In IPM programs, economic thresholds are used to determine whether fumigation is required (Flinn et al. 2007), and coordinated programs over large areas are implemented to reduce pest populations and decrease the risk of infestation and re-infestation (Flinn et al. 2003).

Understanding networks of the movement of pests and other contaminants through grain systems can be used to help target risk management by stored-grain companies, extension personnel, wheat producers (including precision agriculture applications), the milling industry, and government agencies. Network models have proven useful in a wide range of other disciplines. For example, they provide insights into factors that influence individual and collective behavior, the dynamics of the transmission of infectious diseases, and the conservation of endangered species (May 2006, Newman 2010, Chadès et al. 2011, Pautasso and Jeger 2014). Network applications in cropping systems range from seed exchange among farmers to decision support systems, plant disease management, and plant disease epidemiology (Chadès et al. 2011, Garrett 2012, Shaw and Pautasso 2014), where network metrics that identify key nodes can guide efficient sampling for epidemic surveillance (Sutrave et al. 2012).

Here, we provide a new perspective on the structure of networks of wheat movement (see box 1) by evaluating the rail transport network among US states and in two networks within Qld: the *Qld-frequent* network (the most common), in which grain movement is strictly toward ports and there is no grain movement among the main exportation ports, and the *Qld-rare* (which represents unusual years), in which there is a single network and grain moves among all ports. Our goal is to identify critical control points (nodes) for the sampling and mitigation of contaminants moving through these stored-grain networks and to illustrate how this approach could be applied to any stored food and postharvest networks. We study the rail network because rail transport is particularly important for wheat (72% of US wheat was moved by rail in 2001–2010; Prater et al. 2013b) and because of data accessibility. Mycotoxins, pest and fungal populations, pesticide-resistant subpopulations, and other quality problems are common contaminants moving through food networks. There is a need for biosecurity strategies to limit the spread of contaminants, regardless of whether contaminants are accidentally or intentionally introduced into food networks. Network structures can be used to optimize trace-back and trace-forward analyses, identifying nodes where a problem likely originated, the nodes through which it was transferred, and the nodes to which a contaminant may have moved. The intuitive characteristics of stored-grain networks also make them useful case studies, illustrating how network structures can be an integral part of IPM strategies in general and how network analyses can be applied to food, agricultural, epidemiological, and other management systems.

**Box 1. The storage and movement of grain.**

The decision to store grain versus selling is based on the assumption of higher profit in the future; storage allows the owner to forego selling grain when supply is high (at harvest) and market prices are low and to simply store the grain until the price is higher. One of the first grain elevators in the United States was built in Buffalo, New York, in 1842 (Reed 1992). Many “country” or regional elevators served a geographic area small enough that grain could be delivered at harvest by horse and wagon one to several times during a working day (Bailey 1992). Truck transportation broadened the area served by individual country elevators, and these regional elevators continue to serve as a place for farmers to deliver grain during harvest. As an alternative to storage in regional elevators, some farmers with adequate facilities store grain on the farm. Grain moves from country elevators to larger regional elevators. A *terminal elevator* is typically a facility where grain is received from multiple regional elevators within a day of travel. Despite the name *terminal elevator*, grain transported to terminal elevators in the United States rarely terminates its movement at that site. Wheat grain may move several more times from a terminal elevator before reaching its end use at flour mills or being loaded for shipment to major population centers in the United States or elsewhere. Some states export all of the grain they produce, whereas others consume all of the grain they produce internally. *Export elevators*, located in most major US coastal cities, are large terminal elevators that accumulate grain, often from many regions, for export via ship to other countries. Our analysis focuses on rail movement, although movement by truck is particularly important at smaller scales. There is also extensive barge transport of US wheat on the Missouri and Mississippi Rivers in the Central United States and the Columbia River in the Northwest, which may effectively remove wheat from the terrestrial network.

Wheat storage networks rely on effective regional transportation systems, including trucks, railroads, and barges, coupled with permanent structures at key locations. In the United States, networks are facilitated mostly by the private sector, and buyers and sellers know the location, quality, and price of available grain. The risk of pest infestation and fungal proliferation increases with storage time. Grain marketing decisions—for everyone from the farmer who produced the grain to the terminal elevator accumulating thousands of tons of grain—are based on expected profits. Consequently, the impact of mycotoxins and pests often is not considered by end-use markets until their presence is discovered and the problems have caused a significant loss in price or quality.

In many regards, the storage and movement of wheat in Australia is similar to that in the United States, with some major differences. In Australia, the storage and movement of grain are heavily focused on export, although in some parts of the country, there is strong domestic demand, such as from the beef industry. Wheat can be stored by farmers, several large bulk-handling companies, or by many smaller grain companies. Transport is primarily through road and rail, with the major bulk-handling companies maintaining depots along road and rail routes which lead to coastal export facilities.

The objectives of the present work are to (a) evaluate the network structure for stored-wheat rail transport in the United States and Queensland, (b) identify critical control points for the sampling and mitigation of contaminants moving through these stored-grain networks, (c) illustrate the potential for stored-grain systems as models for basic ecology and network analyses, and (d) provide a multidisciplinary synthesis of perspectives from grain management, ecology, and network science.

## Arthropods in grain networks

At least 1900 species of arthropods are known to occur in stored grain or to be associated with grain-based foods, and dozens of these are considered actionable pests because they affect the quality of grain in storage (Hagstrum and Subramanyam 2009). These species are found in almost all countries where cereal grains are stored. The most common stored-grain insect species tend to be restricted to reproducing in bulk stored grain and associated situations, and although some (like *Rhyzopertha dominica*) have been found far from these locations, numbers seem to be relatively low, and information about alternative food sources is scarce. (Hagstrum and Subramanyam 2006). Stored-grain pests feed on the starch, fat, and protein of the stored seeds, and their survival and reproduction often increase if particular fungi are present.

**Figure 2.**
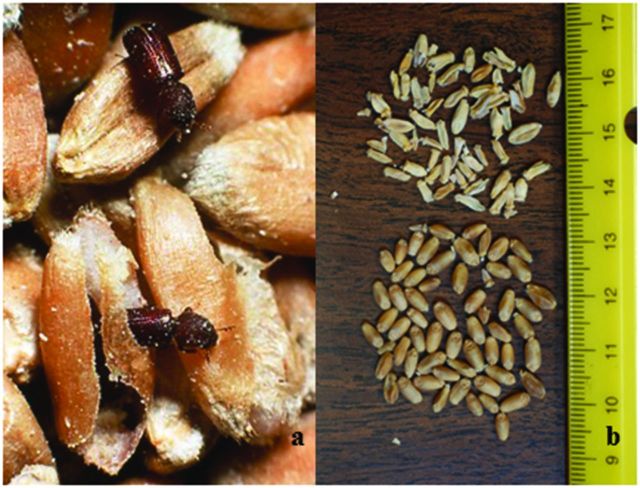
Two common species in stored-grain systems.  (a) The lesser grain borer, Rhyzopertha dominica, a strong flier and serious wheat-damaging insect that is easily transported in the commercial grain-movement network or via natural dispersal behavior. Photograph: Thomas W. Phillips. (b) Wheat kernels. Below: Healthy kernels. Above: Fusarium-damaged kernels, which are at risk of mycotoxin contamination. Photograph: John F. Hernandez Nopsa.

Pests of stored wheat can be categorized by their life histories. Internal feeders have larvae that feed inside grain (Hagstrum et al. 2012), cause significant physical damage to the seed, and are often considered the most serious stored-wheat pests, given that US and Australian wheat is marketed for quality based on the lack of insect damage. Internal feeders are represented by just a few species: the rice weevil (*Sitophilus oryzae*), the maize weevil (*Sitophilus zeamais*), the lesser grain borer (*R. dominica*; figure 2a), and the Angoumois grain moth (*Sitotroga cerealella*). External feeders include dozens of beetles, mites, and moths (Hagstrum et al. 2012). Their larvae feed on broken kernels, grain dust, flour, and fungal spores and mycelia. Particularly important among external feeding grain insects are the red flour beetle (*Tribolium castaneum*) and the rusty grain beetle (*Cryptolestes ferrugineus*). The stored product ecosystem supports multiple trophic levels. Grain is the primary source of nutrition, and most pests feed directly on grain or on mixtures of grain debris and fungi. Parasitic and predatory arthropods feed on many of the pests. Certain vertebrate pests, such as rodents and birds, are also present opportunistically.

Wheat kernels may be infested during or after harvest by pests already present in combine harvesters, trucks, bins, or elevators (Hagstrum et al. 2010). There is no published evidence suggesting that grain pest infestations develop in the field prior to harvest by *S. oryzae* or *R. dominica* in the United States and Australia. However, field infestation of wheat by these species has been reported in other countries (Hagstrum and Subramanyam 2006), and field infestation of wheat by *S. cerealella* has been reported in the United States (Simmons and Ellington 1927). Pests are highly mobile and can be transported through the marketing system whenever grain is moved from one location to another, but low sampling rates and small sample sizes result in relatively few infestations being detected (Hagstrum et al. 2010). In addition to the spread of emerging pathogens and pests, the spread of problematic pest populations, such as pesticide-resistant populations of *S. oryzae, R. dominica*, and *C. ­ferrugineus*, is also an important risk.

Wheat marketing in the United States is graded on the basis of bulk density (i.e., test weight), levels of fine material, the absence of live insects injurious to grain, and the proportion of kernels damaged by pests, among other factors. Therefore, common external feeders, such as *T. castaneum* and *C. ferrugineus*, and the common and very serious internal feeders, such as *R. dominica* and *S. oryzae*, have been the focus of much recent research on IPM. The United States is not only one of the largest producers of wheat in the world but also, along with Australia, one of the largest exporters in the world. Therefore, scientists from these countries have partnered in several collaborations as part of the Australian Plant Biosecurity Cooperative Research Center, including the application of network analyses to refine management strategies for key pests of stored wheat.

## Fungi and mycotoxins in grain networks

Fungi in stored grain not only damage grain but are also serious threats to food safety when they contaminate the grain with mycotoxins in the field and during storage (Birzele and Prange 2003, Magan et al. 2010, Mylona et al. 2012). Both fungi and their mycotoxins can move with stored grain. These fungal toxins must be ingested to have toxic effects, with the exception of T-2, which is toxic upon contact. Mycotoxins in stored wheat include ergot alkaloids (produced by *Claviceps purpurea*), Ochratoxin A (produced by *Aspergillus ochraceus* and *Penicillium verrucosum*, among others), deoxynivalenol (DON) and zearalenone (produced by *Fusarium graminearum* and *Fusarium culmorum*, among others), and T-2 and HT-2 toxins (produced by *Fusarium langsethiae* and *Fusarium sporotrichioides*, among others; Leslie and Summerell 2006, Magan et al. 2010). Wheat kernels affected by Fusarium head blight are referred to as *Fusarium-damaged kernels* (FDK) or *tombstone kernels* (figure 2b). Chalky, shriveled, lightweight kernels covered with white or pink mycelia typify FDK (Parry et al. 1995, Hernandez Nopsa et al. 2012).

The fungus *Tilletia indica*, which causes Karnal bunt of wheat, is subject to strict quarantine sanctions in the United States and other countries. Fungal spores can disperse long distances by wind, but *T. indica* is subject to an *Allee effect* (the reduced probability of reproduction per capita when populations are small) at the edge of invasion zones, such that after wind dispersal, it must encounter individuals of other mating types to reproduce (Garrett and Bowden 2002). Stored grain with fungal proliferation represents a major source of spores, mycelia, and mycotoxins that can readily move with grain in commerce, bypassing the need for fungi such as *T. indica* to survive long-distance wind transport. The 2015 detection of wheat flag smut (caused by *Urocystis tritici*) in several Kansas counties is an example of the motivation to understand networks of stored-wheat movement within states. This pathogen, subject to import restrictions in some countries, can also be transmitted through contaminated seed.

Fungi and pests interact as they move through stored-grain networks. Fungal spores were commonly found attached to the bodies of insects collected from a grain-storage facility in Brazil (Birck et al. 2006), suggesting that insects function as dispersal agents for the fungi. Stored grain respires (Magan et al. 2010), and in longer-term storage, respiration can result in conditions favorable to fungal growth and development and insect population increase. Improper storage conditions can increase the level of DON and ochratoxin A (Birzele and Prange 2003). Harvesting grain at the proper moisture content, regular aeration, appropriate storage conditions, temperature and humidity control, sampling and early detection, as well as other on-farm and storage management practices are important to avoid increases in fungal growth and mycotoxin contamination during storage.

## Integrated pest management

Marketing, wheat type, and available transportation determine the pattern of grain storage and movement. The blending and commingling of grain as it moves through the marketing system can spread pest and pathogen populations (Hagstrum et al. 2010). When more than one crop species is stored at an elevator or carried in a transportation vehicle, pests or pathogens also may move among different types of grain, potentially spreading pesticide resistance.

Currently, the gaseous fumigant insecticide phosphine, or hydrogen phosphide (PH_3_), is the predominant pesticide option for controlling pests in dry commodities and stored products (Hagstrum et al. 1999, Opit et al. 2012). This fumigant has been used for many decades, and resistance has evolved in at least 11 stored-grain pest species in numerous countries, probably from misuse (Boyer et al. 2012). The genetic basis for resistance has only recently been discovered (Schlipalius et al. 2012). Resistance is now a serious threat for the stored-grain industry and a significant threat to food security (Opit et al. 2012, Nayak et al. 2013, Daglish et al. 2014). Resistance has also developed in some species to grain protectant insecticides, which are used for the long-term protection of wheat (Lorini and Galley 1999, Daglish et al. 2013).

Particularly alarming is the emergence of high-level phosphine resistance. This has been recorded in *C. ferrugineus* in Eastern Australia and seems to be related to the long-term storage of grain in grain depots, with lower levels of resistance detected in grain stored on farms, where fumigant use is less common and generally shorter (Nayak et al. 2013). The key components of a plan to eradicate populations of *C. ferrugineus* resistant to phosphine in Australia (Nayak et al. 2010) are (a) pest sampling; (b) resistance testing; (c) pest monitoring, inspection, and an intense hygiene program; and (d) the use of residual insecticide grain protectants and the fumigant sulfuryl fluoride, a phosphine replacement.

Pesticide-resistant pests are commonly moved with grain as it is transported for sale or storage. For example, rice with phosphine-resistant *R. dominica* was imported into Sri Lanka from Pakistan (Tyler et al. 1983), and phosphine-resistant *C. ferrugineus* was intercepted in the United States on commodities imported from India (Dyte and Blackman 1970, Dyte and Halliday 1985). Reports of phosphine resistance have accumulated in both the United States and Australia for *T. castaneum*, *C. ferrugineus, S. oryzae*, and *R. dominica* (Opit et al. 2012) and for *Liposcelis bostrychophila* in Australia (Nayak and Collins 2008). Understanding the transport networks through which pesticide-resistant pests move will provide a foundation for the better management of pesticide resistance. The identification of nodes where fumigation was not successful is important so that these nodes can be treated again to avoid the dispersal of potentially resistant pests to nodes free of resistance. In analyses consistent with the importance of network structure, highly infested bins and bins with pests were observed to be closer to each other (Flinn et al. 2010), and the number of infested bins was observed to decrease with distance from highly infested bins (Hagstrum et al. 2010).

In the United States, the Grain Inspection, Packers, and Stockyards Administration–Federal Grain Inspection Service defines wheat as *infested* when two or more live insects injurious to grain are found per kilogram (kg) of wheat. *Sample-grade* wheat is designated when more than 32 insect-damaged kernels per 100-gram (g) sample are detected. Grain in these grades cannot be sold for human consumption, nor can flour with more than 75 insect fragments per 50 g (Hagstrum and Subramanyam 2006, GIPSA and FGIS 2013). Insects normally are detected when their numbers reach about one insect per kg because of the generally low number of sample units evaluated. During storage, grain infestation occurs in all types of storage sites containing wheat, as well as in empty structures or outside storage units. Once infestation reaches a critical density (more than 2 insects per kg of grain), fumigation is necessary (Flinn et al. 2010, Hagstrum et al. 2010). In Australia, there is zero tolerance for live insects in grain for export, and this standard is often used in domestic markets.

Good sampling is essential to detect mycotoxins, given the heterogeneous distribution of contaminated grain within grain lots. For mycotoxin analysis, sampling error in assessing contamination levels is a large part of the total model error and was estimated to range from 25% to 60% (Magan et al. 2010). Mixing high-quality grain with low-quality grain (e.g., grain with high mycotoxin levels, damaged kernels, or insect-infested grain), also called *blending*, is prohibited by regulatory agencies in the United States for mycotoxins, with some exceptions (Delwiche et al. 2005), although blending probably often occurs unintentionally because of inadequate sampling. The movement of grain contaminated with mycotoxins through transportation networks can limit or prevent grain imports and exports because of international phytosanitary halts and regulations.

## Methods: Analysis of stored-grain networks in the United States and Australia

We identified key nodes that act as major sources, sinks, and bridges for stored-grain movement and therefore constitute high-risk nodes for the movement of associated fungi and pests. The study includes the 37 US states most actively involved in sending or receiving wheat by rail. US state rail receipt information by origin and destination state and information about rail shipments from states to “business economic areas” (often export ports or areas with milling industries; supplemental figure S1) were obtained from the State Grain Rail Statistic Summary for 2006–2010 (Prater et al. 2013a). States with less than three observation years during the 5-year period were excluded by Prater and colleagues (2013a). The volume of wheat moved directly between each pair of states was taken as the link weight in a directed network. For the Qld networks, we included the sites involved in the reception and storage of wheat that are linked by rail in this region. Information about rail linkages and the frequency of wheat movement was from GrainCorp Australian operations. The Qld data lacked grain volume, so the adjacency matrices indicated the presence or absence of a link between nodes in a directed network.

We evaluated a number of traits of nodes in the rail networks to identify nodes at which sampling and management may be particularly important. Node degree gives the number of links for a state, and node strength gives the sum of link weights (i.e., volume of grain transported). For the United States, the adjacency matrix for state-to-state rail movement was evaluated, including the incoming and outgoing node strength and node degree for each state, the shortest path (the minimum number of links separating any two nodes) between each pair of states, betweenness centrality (the number of shortest paths going through a node), and the states present “upstream” and “downstream” from each node. Wheat production (Prater et al. 2013a) was used as one measure of the importance of state nodes in the network. For the Qld rail networks, two adjacency matrices based on geographic location and proximity among storage sites were evaluated. Storage sites that were nearest neighbors on rail lines were assumed to be directly connected, and the direction of grain movement was from storage sites in the countryside toward the port elevators. We evaluated the Qld-frequent (no links among ports) and the Qld-rare (links among ports) network. The Qld analysis was similar to the US analysis but did not include an evaluation of node strength because of the lack of grain-volume data. The R programming environment (R Core Team 2014), including the igraph package, version 0.7.0 (Csardi and Nepusz 2006), was used to evaluate the rail transport networks.

## The structure of stored-grain networks in the United States and Eastern Australia

**Figure 3.**
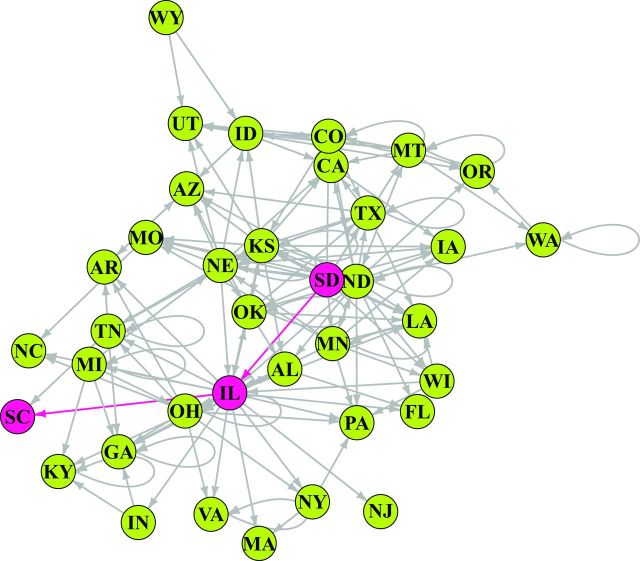
The network of state-to-state wheat movement by rail in the United States. The nodes are states sending or receiving wheat, and the link arrows indicate the directionality of the movement. The red nodes indicate the shortest path from South Dakota to South Carolina, via Illinois. Abbreviations: AL, Alabama; AR, Arkansas; AZ, Arizona; CA, California; CO, Colorado; FL, Florida; GA, Georgia; IA, Iowa; ID, Idaho; IL, Illinois; IN, Indiana; KS, Kansas; KY, Kentucky; LA, Louisiana; MA, Massachusetts; MI, Michigan; MN, Minnesota; MO, Missouri; MT, Montana; NC, North Carolina; ND, North Dakota; NE, Nebraska; NJ, New Jersey; NY, New York; OH, Ohio; OK, Oklahoma; OR, Oregon; PA, Pennsylvania; SC, South Carolina; SD, South Dakota; TN, Tennessee; TX, Texas; UT, Utah; VA, Virginia; WA, Washington; WI, Wisconsin; WY, Wyoming.

The role of each location in the stored-wheat rail networks was evaluated using several network metrics, each capturing a different aspect of the risk of pest or contaminant movement through a node. Thirty-seven nodes (states) were modeled as a directed network of stored-wheat movement by rail in the United States (figure 3). Nodes with high out-degree (and high out-strength) were observed in the US network, but out-degree was low in the Qld network (supplemental table S2, supplemental table S3). The states with the highest incoming node degree—that is, important sinks (recipient states) for grain along with potential grain pests or contaminants—were Louisiana, which is connected to eight source states, and Arizona, Georgia, and Texas, each of which is connected to seven source states (supplemental table S1). The states with high incoming node strength were Texas, Illinois, Washington, Oregon, and Wisconsin (table S2). The states with zero incoming node degree were South Dakota and Wyoming (table S1), and the states with near-zero incoming node strength were Arkansas, Idaho, Kentucky, and Montana (table S2).

The states with the highest outgoing node degree—that is, important sources of grain and potentially contaminants—were North Dakota, Illinois, Kansas, and Nebraska, which were connected to 20, 17, 14, and 13 sink states, respectively (table S1). Additional states with high outgoing node strength, such as Montana and South Dakota, are also generally in the central part of the United States and produce large amounts of wheat (table S2). The states with zero outgoing node degree for rail transport were Arizona, Florida, Iowa, Louisiana, Massachusetts, North Carolina, New Jersey, Oregon, Pennsylvania, South Carolina, and Utah (table S1), along with nine other states having near-zero outgoing node strength (table S2).

States that are part of the shortest path between other pairs of states may play key roles in the spread of pests and fungi by bridging different sections of the network (Newman 2010). For example, South Dakota is connected to South Carolina in two steps, via Illinois (figure 3). Likewise, Kansas is connected to 13 states in a single step and to 16 states in two steps, and South Dakota is connected to 12 states in one step and to 23 states in two steps. States that are “upstream” from many other states are likely important in the rapid dispersal of pest and diseases through the network (e.g., South Dakota, Minnesota, Montana, and North Dakota; table 1). States that are “downstream” from many states are more likely to receive pests and pathogens (e.g., Arizona and Georgia, among others; table 1). The average shortest path length between two nodes in the US network is 2.08. Potentially the most important nodes for the spread of contaminants are Illinois, Kansas, Colorado, Idaho, Nebraska, and North Dakota, because these nodes have the highest values of *betweenness centrality* (the number of shortest paths crossing a node; table 2). This information about risk can be used in two general contexts: preoutbreak and postoutbreak. Before an outbreak, states that have high numbers of shortest paths going through them should be priorities for sampling and surveillance to prevent or limit the spread of pests, pathogens, and contaminants. Once a problem is detected in the system, network analysis can identify the priority nodes and the paths for mitigation linked to the contaminated nodes. The origin of the problem can also be traced backward—and its movement traced forward through the network—to adapt management strategies.

**Table 1.** State roles as source or sink in rail transport of wheat in the United States.

	Number of sites connected to focal site	
Focal state	Focal state as source	Focal state as sink
AZ, GA	0	17
FL, IA, LA, MA, NC, PA, SC	0	15
KY, OR, VA	0	14
NJ, UT	0	13
AR	1	15
CA	1	14
MO	1	13
WA	1	4
AL, NY	2	14
IN	2	13
TN	4	14
TX	4	13
OH	13	13
CO, ID, IL, KS, MI, NE, OK	29	12
WI	30	4
WY	30	0
MN, MT, ND	34	3
SD	35	0

*Note:* The source or sink status indicates whether the focal state is “upstream” or “downstream,” respectively. Abbreviations: AL, Alabama; AR, Arkansas; AZ, Arizona; CA, California; CO, Colorado; FL, Florida; GA, Georgia; IA, Iowa; ID, Idaho; IL, Illinois; IN, Indiana; KS, Kansas; KY, Kentucky; LA, Louisiana; MA, Massachusetts; MI, Michigan; MN, Minnesota; MO, Missouri; MT, Montana; NC, North Carolina; ND, North Dakota; NE, Nebraska; NJ, New Jersey; NY, New York; OH, Ohio; OK, Oklahoma; OR, Oregon; PA, Pennsylvania; SC, South Carolina; SD, South Dakota; TN, Tennessee; TX, Texas; UT, Utah; VA, Virginia; WA, Washington; WI, Wisconsin; WY, Wyoming.

**Table 2.** Betweenness centrality (defined as the number of shortest paths going through a node) in 37 states involved in wheat movement by rail in the United States (2006–2010).

Node	Betweenness centrality
IL	145
KS	98
CO	46
ID	44
NE	32
ND	31
OK	13
OH, MI	9
AL, TN	8
MO, TX	5
CA, MT	4
AR	3
NY	2
MN	1
AZ, FL, GA, IN, IA, KY, LA, MA, NJ, NC, OR, PA, SC, SD, UT, VA, WA,WI, WY	0
0	

Abbreviations: AL, Alabama; AR, Arkansas; AZ, Arizona; CA, California; CO, Colorado; FL, Florida; GA, Georgia; IA, Iowa; ID, Idaho; IL, Illinois; IN, Indiana; KS, Kansas; KY, Kentucky; LA, Louisiana; MA, Massachusetts; MI, Michigan; MN, Minnesota; MO, Missouri; MT, Montana; NC, North Carolina; ND, North Dakota; NE, Nebraska; NJ, New Jersey; NY, New York; OH, Ohio; OK, Oklahoma; OR, Oregon; PA, Pennsylvania; SC, South Carolina; SD, South Dakota; TN, Tennessee; TX, Texas; UT, Utah; VA, Virginia; WA, Washington; WI, Wisconsin; WY, Wyoming.

**Figure 4.**
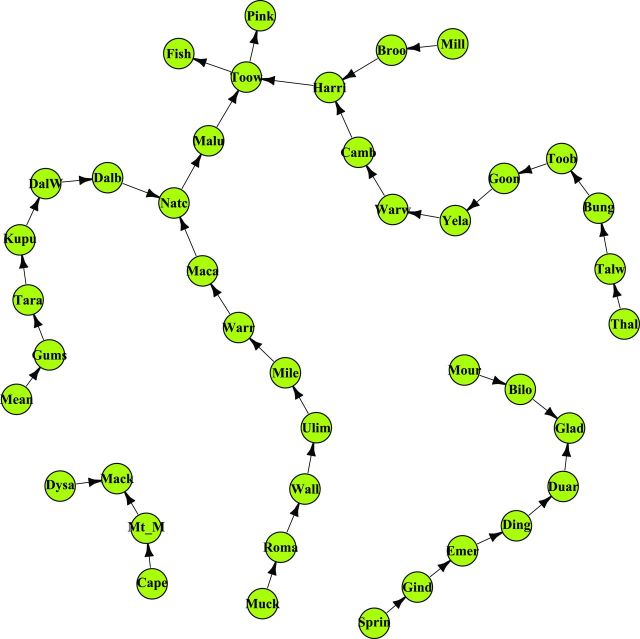
The “Qld-frequent” network of storage sites connected by rail in Queensland (Qld), Australia, representing the network structure when exchange among coastal Qld nodes does not occur. This represents the usual situation. The links indicate the directionality of wheat movement. Abbreviations: Bilo, Biloela; Broo, Brookstead; Bung, Bungunya; Camb, Cambooya; Cape, Capella; Dalb, Dalby; DalW, Dalby West; Ding, Dingo; Duar, Duaringa; Dysa, Dysart; Emer, Emerald; Fish, Fisherman Islands; Gind, Gindie; Glad, Gladstone; Goon, Goondiwindi West; Gums, The Gums; Harri, Harristown; Kupu, Kupunn; Maca, Macalister; Mack, Mackay; Malu, Malu; Mean, Meandarra; Mile, Miles; Mill, Millmerran; Mour, Moura; Mt_M, Mt McLaren; Muck, Muckadilla; Natc, Natcha; Pink, Pinkenba; Roma, Roma West; Sprin, Springsure; Tara, Tara; Talw, Talwood; Thal, Thallon; Toob, Toobeah; Toow, Toowoomba; Ulim, Ulimaroa; Wall, Wallumbilla; Warr, Warra; Warw, Warwick; Yela, Yelarbon. The main ports (Gladstone, Fisherman Island, Pinkenba, and Mackay) are not connected.

**Figure 5.**
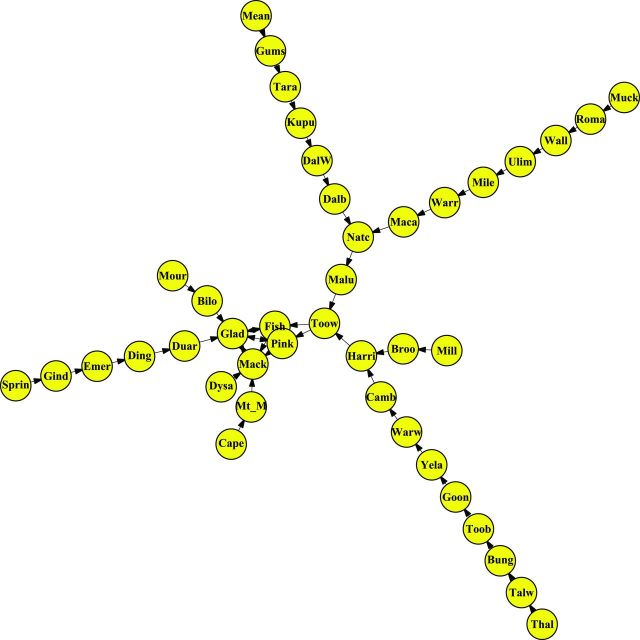
“Qld-rare” network of storage sites connected by rail in Queensland (Qld), Australia, representing the unusual scenario in which there is some exchange among coastal Qld nodes. The links indicate directionality of wheat movement. The site abbreviations are the same as those in figure 4. The main ports (Gladstone, Fisherman Island, Pinkenba, and Mackay) are connected and interchange wheat.

In the Qld-frequent network ­(figure 4), despite the existence of a rail connection, wheat is not transported among the main exportation ports (Fisherman Islands, Port of Brisbane; Gladstone; Mackay; and Pinkenba). The second unusual case is a network in which there is movement of stored grain among these ports ­(figure 5). For the Qld-frequent network, we found three separate subnetworks of different lengths, without movement of grain among the subnetworks. Node degree for Qld-frequent ranged from *0* to *2*. *Transitivity* is a measure of network clustering, in which the minimum transitivity is *0* and a network in which every node is fully connected to all the other nodes in the network has transitivity equal to *1* (Newman 2010). The transitivity of the Qld-frequent network is *0*. These metrics indicate that there is low interconnectivity among nodes. Therefore, the risk of pathogen or pest movement in the network is also low, and in the Qld-frequent scenario, the separate subnetworks have an independent risk of spread through rail networks. Sites such as Fisherman Islands, Gladstone, Mackay, and Pinkenba are the recipient nodes from the subnetworks and so are particularly likely to receive contaminants and may be a focus for surveys, sampling, and management. For the second scenario (Qld-rare network), in which all the main exportation ports are connected, the sites with the highest outgoing node degree were Fisherman Islands, Gladstone, and Pinkenba, whereas the sites with high incoming node degree were Gladstone and Mackay, which are connected to five source sites, followed by Fisherman Islands and Pinkenba, each of which is connected to three sites (figure 5, table S3). The sites with the highest incoming node degree are important sinks (recipient sites) for grain along with potential grain pests or contaminants. The locations with the lowest (zero) incoming node degree were Capella, Dysart, Springsure, Moura, Muckadilla, Meandarra, Millmerran, and Thallon (table S3).

The Qld sites “upstream” from the greatest number of nodes were Muckadilla and Thallon. The sites “downstream” from the greatest number of nodes were the four coastal export terminals of Fisherman Islands (Port of Brisbane), Gladstone, Mackay, and Pinkenba (table 3). The key nodes based on betweenness centrality were Toowoomba, Natcha, Malu, and Harristown in both the frequent and rare networks (table 4). Toowoomba has a technical laboratory, and many samples may arrive from other nodes in the network for analysis and certification, increasing the risk of pest and contaminant traffic through this node. Gladstone, a port elevator, is a source and sink for many nodes. These four nodes are important for the potential movement of pests in the Qld network and are priorities for sampling and surveillance. The average shortest path between two nodes for the Qld-rare network was *4.3*, about twice the larger state-level US rail network but very similar to the Qld-frequent network (*4.0*).

**Table 3.** Site roles as source and sink in the movement of wheat among storage sites by rail in Queensland, Australia, through the shortest paths between storage sites.

	Number of sites connected to focal site	
Focal site	Focal site as source	Focal site as sink
Gladstone, Fisherman Islands, Mackay, Pinkenba	3	40
Toowoomba	4	26
Malu	5	14
Natcha	6	13
Harristown	5	10
Cambooya	6	7
Macalister, Warwick	7	6
Warra, Yelarbon	8	5
Dalby	7	5
Miles, Goondiwindi West	9	4
Dalby West	8	4
Duaringa	4	4
Toobeah, Ulimaroa	10	3
Kupunn	9	3
Dingo	5	3
Bungunya, Wallumbilla	11	2
Tara	10	2
Emerald	6	2
Roma West, Talwood	12	1
The Gums	11	1
Gindie	7	1
Brookstead	6	1
Mt. McLaren	4	1
Biloela	4	1
Muckadilla, Thallon	13	0
Meandarra	12	0
Springsure	8	0
Millmerran	7	0
Capella, Moura	5	0
Dysart	4	0

*Note:* The source or sink status indicates whether the focal state is “upstream” or “downstream,” respectively.

**Table 4.** Betweenness centrality (defined as the number of shortest paths going through a node) in 41 sites connected by rail in Queensland (Qld), Australia.

	Betweenness centrality	
Node	Qld-rare	Qld-frequent
Toowoomba	104	52
Natcha	78	52
Malu	70	42
Harristown	50	30
Macalister, Warwick	42	30
Cambooya	42	28
Warra, Yelarbon	40	30
Goondiwindi West, Miles	36	28
Dalby	35	25
Dalby West	32	24
Ulimaroa, Toobeah	30	24
Gladstone	29	0
Fisherman Islands, Pinkenba	27	0
Kupunn	27	21
Bungunya, Wallumbilla	22	18
Tara	20	16
Duaringa	16	4
Dingo	15	6
Roma West, Talwood,	12	10
Emerald	12	6
The Gums	11	9
Mackay	9	0
Gindie	7	4
Brookstead	6	4
Biloela, Mt McLaren	4	1
Capella, Dysart, Meandarra, Millmerran	0	0
Moura, Muckadilla, Springsure, Thallon	0	0

*Connectance*, the number of links divided by the total possible number of links, was *0.11* in the US network (901 pairs of nodes unconnected), 3.6 times higher than the connectance in the Qld-rare network (*0.03*, with 1340 pairs of nodes unconnected) and 5.5 times higher than in the Qld-frequent network (*0.02*, with 1436 pairs of nodes unconnected). The US rail network transitivity is *0.38*. The Qld-rare network transitivity was *0.21*, similar to the value obtained for the US rail network and very different from the Qld-frequent network (*0*).

In the US network, there were 135 shortest paths that consisted of a single link, 2.8 and 3.6 times higher than in the two Qld networks (table 5). Also, the longest shortest path in the US network had 5 links, compared with the longest in the Qld networks, which had 11 and 10 links. The Qld-rare network had a longer maximum shortest path than the Qld-frequent network, reflecting the fact that all nodes in Qld-rare were included in a single network (table 5).

**Table 5.** The number of times each shortest path length was observed in the US and Queensland (Qld) networks.

	Shortest paths		
Shortest path length (number of links)	US	Qld-rare	Qld-frequent
1	135	48	38
2	185	46	34
3	69	45	30
4	27	38	27
5	15	32	21
6	–	26	17
7	–	20	14
8	–	17	11
9	–	14	8
10	–	10	4
11	–	4	–
No path exists	901	1340	1436

**Figure 6.**
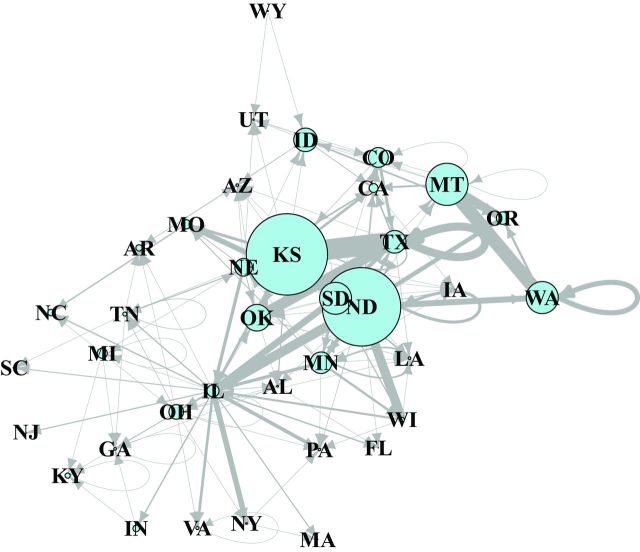
A network depicting both wheat movement (links) and production (nodes) in the United States in 2006–2010. The link thickness is proportional to the amount of wheat transported, and the node size is proportional to the volume of wheat produced by each state. The state abbreviations are the same as those in figure 3.

Additional visual perspective on the structure of the US stored-wheat network can be gained by modifying the network representation. When link width was modified to be proportional to the volume of wheat sent among states, the importance of Illinois and Kansas is highlighted (figure S4). When link weight represents the amount of wheat transported and wheat production per state is proportional to node size, the network reflects the importance of Kansas, North Dakota, Montana, Washington, and South Dakota as the largest wheat producers in the United States (figure 6).

## Implications of network structure for management

A striking difference between the US and Qld grain networks is that betweenness centrality is highest for *coastal* depots in the Qld network, whereas betweenness centrality is highest for *central* US states. Of course, the different scales of consideration—states as nodes versus depots as nodes—must also be taken into account in US–Qld comparisons. The differences between US and Qld networks have important implications for the management strategies of both systems. Sampling and mitigation strategies within the US network should emphasize locations in the Central United States, from which contaminants are most likely to move widely through the system. In Qld, sampling and mitigation in the coastal depots and a moderate distance upstream are probably the most efficient for detecting the spread of pests and pathogens. In both the US and Qld systems, sampling and mitigation at ports of debarkation are critical to ensure that materials satisfy international requirements for export. For local analyses, network motifs (Chadès et al. 2011) may be useful for targeting critical nodes for sampling and management.

There is great potential for using information about stored-grain network structure to support pest and pathogen management and to enhance food security. We analyzed average network conditions to identify locations that are commonly highly connected. If a problem such as pesticide resistance or mycotoxin contamination is detected, then detailed information about recent network activity can be used to optimize sampling and mitigation strategies. Network models also can be used to estimate the impact of particular management strategies, such as pesticide use, or the level of pesticide resistance. For example, given information about recent network structure and the selection pressure resulting from pesticide use at each node, the efficacy of different pesticide-use policies can be evaluated. Analyses of global networks for stored grain could be used to assess the risk of invasion by exotic pests, such as the potential for movement of *Trogoderma* sp. into Australia.

Sampling for pesticide resistance can be particularly challenging. The inheritance of phosphine resistance in grain beetles is almost completely recessive (Collins et al. 2002, Daglish 2004, Jagadeesan et al. 2012). Discriminating dose tests usually are effective for detecting homozygous resistant individuals but are less effective for detecting heterozygotes, which means that such tests may fail to detect and characterize resistance threats. Selection for phosphine resistance can occur anywhere that phosphine is used (farms or other nodes) and would first appear in heterozygotes. The movement of resistance alleles via pest dispersal influences how quickly resistant populations develop and spread. The purchase of grain by farmers may introduce resistance to farms. Understanding network structures will help identify nodes linked to locations where heavy selection pressure has occurred and nodes vulnerable to the results of selection pressure at many nodes. Resistance can then be anticipated and resistance management tactics implemented to reduce selection pressure in key nodes.

## The ecology of stored-grain networks

Network models offer an important perspective on spatial ecology, but there are also sometimes significant problems in application, such as difficulty in delineating patches (nodes) and connections (links) and in identifying thresholds that define the presence or absence of nodes or links (Moilanen 2011). Stored-grain networks are unique ecological systems in which nodes are generally well defined (bins, silos, elevators), as are the transportation connections among them. Compared with knowledge of other commercial systems (e.g., ornamental and horticultural networks, in which the structure is largely undocumented; Pautasso and Jeger 2014), relatively more information is available about stored-grain movement in the United States and Australia. Therefore, stored-grain networks have great potential as model systems for the study of dispersal and gene flow in ecological networks.

Many aspects of stored-product pest ecology remain unknown, including the quantification of the contributions of passive transport with grain and of active dispersal through flight. The magnitude of movement in either direction between native vegetation and stored grain remains to be characterized. The relative importance of active and passive movement undoubtedly differs across scales, with active movement becoming less important as distance increases. The movement of pests by grain transport has been demonstrated for key pests (Perez-Mendoza et al. 2004) and is particularly important for sessile stages, such as eggs, pupae, and larvae. Grain is often transported in open hopper cars, so opportunities exist for quarantine pests to escape from infested grain in the transportation network (Perez-Mendoza et al. 2004). Flight dispersal has been documented for some pest species in grain (Edde et al. 2006, Mahroof et al. 2010, Ridley et al. 2011b). Data suggest that pests can fly out of storage structures, and *R. dominica* and *T. castaneum* have been trapped in native vegetation areas away from storage structures (Mahroof et al. 2010, Ridley et al. 2011b). Network modeling at different scales provides the potential to generate hypotheses about the role of human movement of grain, pests, and resistance alleles. Departure from expectations may help to identify the role of active dispersal in pests.

A key factor for biota in stored-grain systems is the effect of weather. Unlike fresh fruits and vegetables, grains are durable agricultural products that do not require refrigeration, immediate processing, or packaging for long-term storage. Most of the United States has a temperate climate, and many long-term grain-storage areas have air temperatures at or near freezing for days to weeks during the winter, along with cool temperatures (20 degrees Celsius and below) in autumn and spring in northern states, allowing safe storage. Wheat stored at a cool temperature and with the recommended safe moisture content of 12% can be stored for months or years without needing treatment for pests or fungi (Phillips and Throne 2010). Southern states with hot summers and relatively warm winters are at higher risk for pest infestations. Unfortunately, most grain-storage facilities allow some access by pests and fungi, and wheat sometimes is stored at higher moisture levels and temperatures, which can facilitate the substantial growth of pest and fungal populations. Unlike the United States, wheat growing and storage areas in Australia rarely, if ever, experience freezing or near-freezing temperatures, so conditions for insect development remain favorable for long periods. In a postharvest transportation network, the risk of a node as a source of biotic contaminants is not only a function of connectivity but also a function of the weather conditions within that state at harvest and during storage. More detailed network models could incorporate observations and estimates of the degree of contamination in each node as a function of weather. Climate change may influence the risk of pest and pathogen dispersal in postharvest networks through changes in the geographic distribution of wheat production (Ortiz et al. 2008), the resulting transport network structure, and storage conditions. The evolution of the invasion network, due to shifts in both climate and markets, will likely make it necessary to frequently update network models used for management strategies (Sanatkar et al. 2015).

## Developing world context

There are additional challenges for managing stored grain in developing countries. Tropical countries in which climate predisposes grain to postharvest losses often have limited or inadequate storage and transport infrastructure. On-farm grain-storage facilities in developing countries may be constructed from grass, bamboo, wood, or mud, although the number of small metallic silos is increasing (Yusuf and He 2011). Pests, fungi, and rodents have ready access to many of these structures and often cause significant damage to stored grain. In India, on-farm storage losses in wheat in the state of Karnataka were 22%, the most important cause of farm-level grain loss (Basavaraja et al. 2007). In Tunisia, all samples in a survey of 127 wheat silos contained field and postharvest fungi (Belkacem-Hanfi et al. 2013). The quality of acceptable wheat varies depending on country and procuring agency. The Food Corporation of India buys grain from farmers at a minimum support price (the lowest price that can be paid to farmers), moves the grain through the public distribution system, and stores the grain to ensure food security in India. In addition to standards for biotic contamination, restrictions on moisture levels and damaged, shriveled, and broken grains may limit the movement of pest-infested wheat. However, many of these restrictions are relaxed in the event of crop failure due to adverse weather conditions (FCI 2014).

Pest infestations can also worsen during transport. In Africa, only one-third of roads are paved, so the breakdown of vehicles is not uncommon during the transport of agricultural commodities (Ait-Oubahou 2013). Poor protection of grain and tropical weather with frequent rains can therefore lead to the spread of fungi in grain. In some cases, grain in very poor condition (high levels of mycotoxins or pests) may be used for human consumption (Bankole et al. 2006, Mutiga et al. 2014). The dynamics of wheat movement in developing countries can therefore be quite different than those in developed countries, and they are often more difficult to accurately describe because of limited data. Networks of grain movement in developing countries will tend to have many nodes, when considered at fine spatial resolution, because of the large number of smallholder farmers. Countries that are located in the humid tropics and have limited regulation will tend to have a higher probability of contaminant movement between nodes, making it difficult to contain outbreaks.

## Postharvest networks as a novel context for network modeling

**Figure 7.**
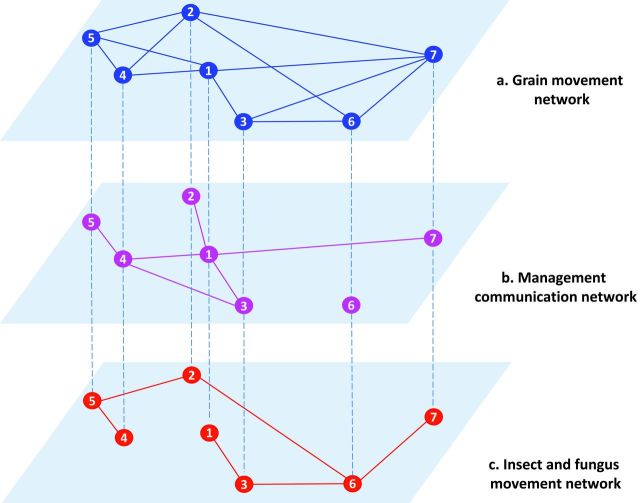
A hypothetical scenario for a multilayer network in the stored-grain system. (a) Stored-grain movement network. The nodes represent stored-grain facilities, and the links represent grain movement among them by rail. (b) A network of communication about management associated with the stored-grain system. All stored-grain facilities (nodes) in the network had access to information about management practices for pests and pathogens except node 6. (c) A pest and fungus movement network. The nodes represent stored-grain facilities, and the links represent the movement of pests and pathogens through the network.

Postharvest networks may be composed of multiple coupled networks. The stored-grain network is interconnected with multiple transport networks at different levels of spatial resolution (e.g., trains moving grain among elevators or trucks transporting grain between farms and elevators) and may be intertwined with a network of active pest movement. Studying the stored-grain rail network in isolation provides an entry point to understanding important network features, but it is not a complete assessment of the system. A new class of problems in network science considers spread through multilayer and interconnected networks. In a multilayer network, the same nodes are connected via *multiple* different link types, each type constituting a specific layer, such as networks of pathogen or pest movement and information about movement and management (Garrett 2012). Consider a hypothetical scenario for a multilayer network of grain, pests and fungi, and communication about management in a stored-grain system (figure 7): With the exception of node 6, all nodes (silos etc.) are linked in a network of communication about management to reduce the risk of insecticide resistance—that is, the application of an appropriate insecticide at the recommended rate and time (figure 7b). A corresponding network of pest movement between nodes (figure 7c) represents passive movement through a subset of the links (e.g., rail transport; figure 7a). The same nodes are simultaneously components of each of the three networks.

**Figure 8.**
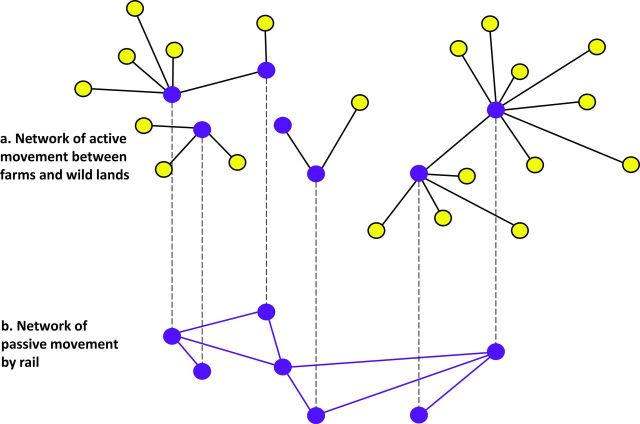
A hypothetical scenario representing an interconnected stored-grain network. (a) A network of active pest movement. The dark nodes are stored-grain structures. The light nodes represent alternative habitat that is suitable for pest survival and development; pests may fly between these and stored-grain structures. (b) A network of passive pest movement. The nodes represent stored-grain facilities, and the links represent rail transportation, supporting passive movement of pests among nodes.

In an interconnected network ­(figure 8), smaller networks, called *components*, may be interconnected with each other (e.g., pests leaving silos and flying to natural environments and pests moved by trains between silos). In this hypothetical scenario, active and passive pest movements form interconnected networks. For example, a network of active pest movement from silos (nodes) to farms or alternative habitats outside of the bulk grain-storage system (figure 8a) may be interconnected with a network of passive movement of pests by rail among silos (figure 8b). In this example of an interconnected network, some—but not all—nodes are shared by different networks. Multilayer and interconnected networks are emerging topics in network science, with numerous potential applications to social, biological, and technological networked systems (Sahneh et al. 2013). Technologies for the improved identification and tracking of batches of grain will enhance the application of network models. As detailed data about the movement of grain become available globally, including in the more diffuse grain-storage and -movement systems of developing economies, the theory of multilayer and interconnected networks will support the identification of vulnerabilities and the development of management strategies.

## Supplementary Material

SUPPLEMENTAL MATERIAL

## Supplemental material

The supplemental material is available online at *http://bioscience.oxfordjournals.org/lookup/suppl/doi:10.1093/biosci/biv122/-/DC1*.
