# Supplementary material for: Ecological Networks in Stored Grain: Key Postharvest Nodes for Emerging Pests, Pathogens, and Mycotoxins
Source: Bioscience. 2015 Sep 9;65(10):985–1002. doi: 10.1093/biosci/biv122 (PMC4718207; doi:10.1093/biosci/biv122)
Supplement: SUPPLEMENTAL MATERIAL [file supp_65_10_985__index.html]

SUPPLEMENTAL MATERIAL 

# Ecological Networks in Stored Grain: Key Postharvest Nodes for Emerging Pests, Pathogens, and Mycotoxins

## SUPPLEMENTAL MATERIAL

- SUPPLEMENTAL MATERIAL
