# Supplementary material for: Ecological Networks in Stored Grain: Key Postharvest Nodes for Emerging Pests, Pathogens, and Mycotoxins
Source: Bioscience. 2015 Sep 9;65(10):985–1002. doi: 10.1093/biosci/biv122 (PMC4718207; doi:10.1093/biosci/biv122)
Supplement: SUPPLEMENTAL MATERIAL [file supp_biv122_suppl_data.zip › Hernandez_Nopsa_et_al_SI_no_figures_v3.docx]

**Supplemental Information**

**Ecological Networks in Stored Grain: Identifying Key Nodes for Emerging Pests and Mycotoxins in Postharvest Networks**

John F. Hernandez Nopsa, Gregory J. Daglish, David W. Hagstrum, John F. Leslie, Thomas W. Phillips, Caterina Scoglio, Sara Thomas-Sharma, Gimme H. Walter, and Karen A. Garrett

**The Supplemental Information includes:**

Supplemental Tables

Supplemental Figures

**Table S1**. Node degree, indicating state roles as source or sink in the movement of wheat by rail in the United States. States are ordered by in-degree.

| Focal state |  | Node degree | | |
| --- | --- | --- | --- | --- |
|  |  | **Out-degree** |  | **In-degree** |
| LA |  | 0 |  | 8 |
| AZ |  | 0 |  | 7 |
| TX |  | 4 |  | 7 |
| GA |  | 1 |  | 7 |
| IL |  | 17 |  | 6 |
| KS |  | 14 |  | 6 |
| NE |  | 13 |  | 6 |
| OK |  | 5 |  | 6 |
| CA |  | 1 |  | 6 |
| IA, PA, UT |  | 0 |  | 6 |
| CO, TN |  | 4 |  | 5 |
| AL |  | 2 |  | 5 |
| MO |  | 1 |  | 5 |
| ID |  | 6 |  | 4 |
| AR, KY |  | 1 |  | 4 |
| OR |  | 0 |  | 4 |
| ND |  | 20 |  | 3 |
| OH |  | 10 |  | 3 |
| MN |  | 9 |  | 3 |
| MT |  | 8 |  | 3 |
| WI |  | 3 |  | 3 |
| WA |  | 2 |  | 3 |
| VA |  | 1 |  | 3 |
| FL, NC |  | 0 |  | 3 |
| MI |  | 5 |  | 2 |
| NY |  | 2 |  | 2 |
| MA, SC |  | 0 |  | 2 |
| IN |  | 2 |  | 1 |
| NJ |  | 0 |  | 1 |
| SD |  | 12 |  | 0 |
| WY |  | 2 |  | 0 |

**Table S2**. Node strength of every state as source or sink in the movement of wheat (million tons in 2006-2010) among states by rail in the United States.

| Focal state |  | Node strength | | |
| --- | --- | --- | --- | --- |
|  |  | **In-strength** |  | **Out-strength** |
| ND |  | 3.52 |  | 46.74 |
| KS |  | 2.71 |  | 34.98 |
| MT |  | 0.37 |  | 23.59 |
| IL |  | 26.59 |  | 23.03 |
| SD |  | 0.00 |  | 16.70 |
| NE |  | 0.71 |  | 12.54 |
| OK |  | 2.34 |  | 11.57 |
| TX |  | 51.71 |  | 9.90 |
| MN |  | 8.28 |  | 9.13 |
| WA |  | 23.74 |  | 7.18 |
| CO |  | 1.05 |  | 3.72 |
| ID |  | 0.24 |  | 3.26 |
| OH |  | 2.19 |  | 3.03 |
| WI |  | 12.31 |  | 2.56 |
| MO |  | 10.99 |  | 1.32 |
| TN |  | 3.76 |  | 1.32 |
| MI |  | 0.66 |  | 0.88 |
| NY |  | 5.76 |  | 0.49 |
| CA |  | 9.22 |  | 0.30 |
| GA |  | 2.07 |  | 0.29 |
| IN |  | 1.33 |  | 0.22 |
| VA |  | 3.27 |  | 0.19 |
| AL |  | 2.47 |  | 0.18 |
| AR |  | 0.15 |  | 0.09 |
| WY |  | 0.00 |  | 0.09 |
| KY |  | 0.26 |  | 0.04 |
| OR |  | 16.01 |  | 0.00 |
| PA |  | 5.34 |  | 0.00 |
| LA |  | 3.83 |  | 0.00 |
| NC |  | 2.84 |  | 0.00 |
| IA |  | 2.00 |  | 0.00 |
| FL |  | 1.49 |  | 0.00 |
| AZ |  | 1.41 |  | 0.00 |
| UT |  | 1.33 |  | 0.00 |
| MA, SC |  | 1.16 |  | 0.00 |
| NJ |  | 1.10 |  | 0.00 |

**Table S3**. Node degree indicating site roles as source and sink in the movement of wheat among storage sites by rail in Queensland, Australia.

| Focal site |  | Node degree | | |
| --- | --- | --- | --- | --- |
|  |  | **Out-degree** |  | **In-degree** |
| Gladstone |  | 3 |  | 5 |
| Mackay |  | 1 |  | 5 |
| Fisherman Islands, Pinkenba |  | 3 |  | 3 |
| Toowoomba |  | 2 |  | 2 |
| Harristown, Natcha |  | 1 |  | 2 |
| Biloela, Bungunya, Brookstead, |  | 1 |  | 1 |
| Cambooya, Dalby, Dalby West, |  | 1 |  | 1 |
| Dingo, Duaringa, Emerald, Gindie, |  | 1 |  | 1 |
| Goondiwindi West, The Gums, |  | 1 |  | 1 |
| Kupunn, Macalister, Malu, Mt. |  | 1 |  | 1 |
| McLaren, Miles, Roma West, |  | 1 |  | 1 |
| Talwood, Tara, Toobeah, Ulimaroa, |  | 1 |  | 1 |
| Wallumbilla, Warra, Warwick, |  | 1 |  | 1 |
| Yelarbon |  | 1 |  | 1 |
| Capella, Dysart, Springsure, Moura |  | 1 |  | 0 |
| Muckadilla, Meandarra, Millmerran, |  | 1 |  | 0 |
| Thallon |  | 1 |  | 0 |

**Figure Captions**

Fig. S1. Network of state-to-business economic areas (BEA) for wheat movement by rail in the United States. Yellow nodes represent US states and blue nodes represent ports and other final destinations for grain stored within the states. State abbreviations are as in Fig. 3. MX.1 represents a port in Mexico.

Fig. S2. Node degree distribution (the frequency distribution of the number of links per node) for stored grain movement by rail in the United States and the "rare" network in Queensland ("Qld-rare"), Australia, that may occur when there is an unusual amount of exchange among coastal Qld nodes. Incoming and outgoing links are shown. The greater abundance of higher-degree nodes in the US network indicates greater potential for spread of contaminants via rail among US states compared to Qld locations.

Fig. S3. Node strength (the sum of link weights incident to a given node) for stored grain movement by rail in the United States (million tons in 2006-2010). Incoming and outgoing strength is shown.

Fig. S4. Network of state-to-state wheat movement by rail in the United States. Link thickness is proportional to the volume of wheat sent among states and its directionality. State abbreviations are as in Fig. 3.
