# Supplementary figures and images for: Ecological Networks in Stored Grain: Key Postharvest Nodes for Emerging Pests, Pathogens, and Mycotoxins
Source: Bioscience. 2015 Sep 9;65(10):985–1002. doi: 10.1093/biosci/biv122 (PMC4718207; doi:10.1093/biosci/biv122)

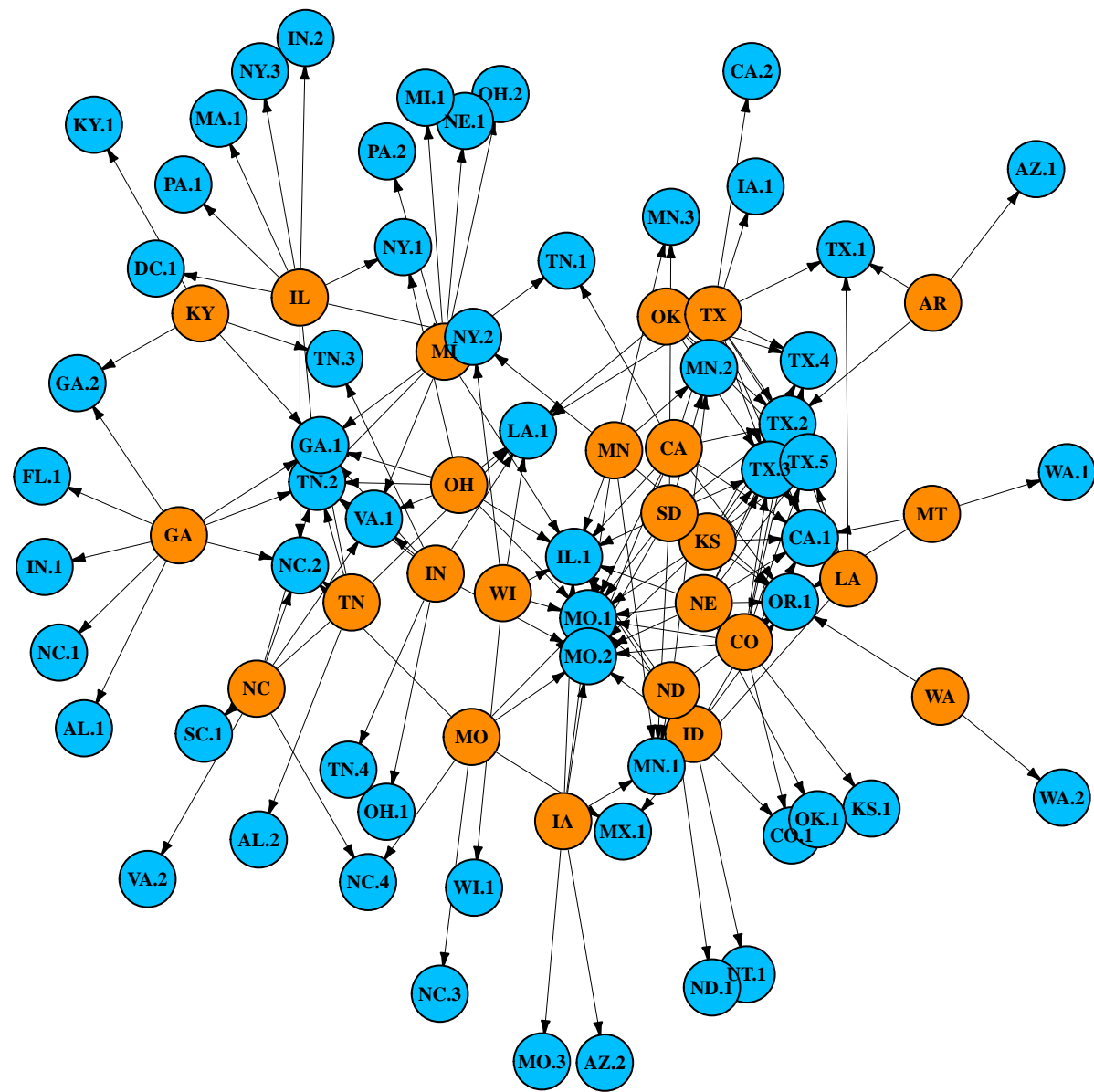

Supplement: SUPPLEMENTAL MATERIAL [file supp_biv122_suppl_data.zip › BioScience Fig. S1.pdf]

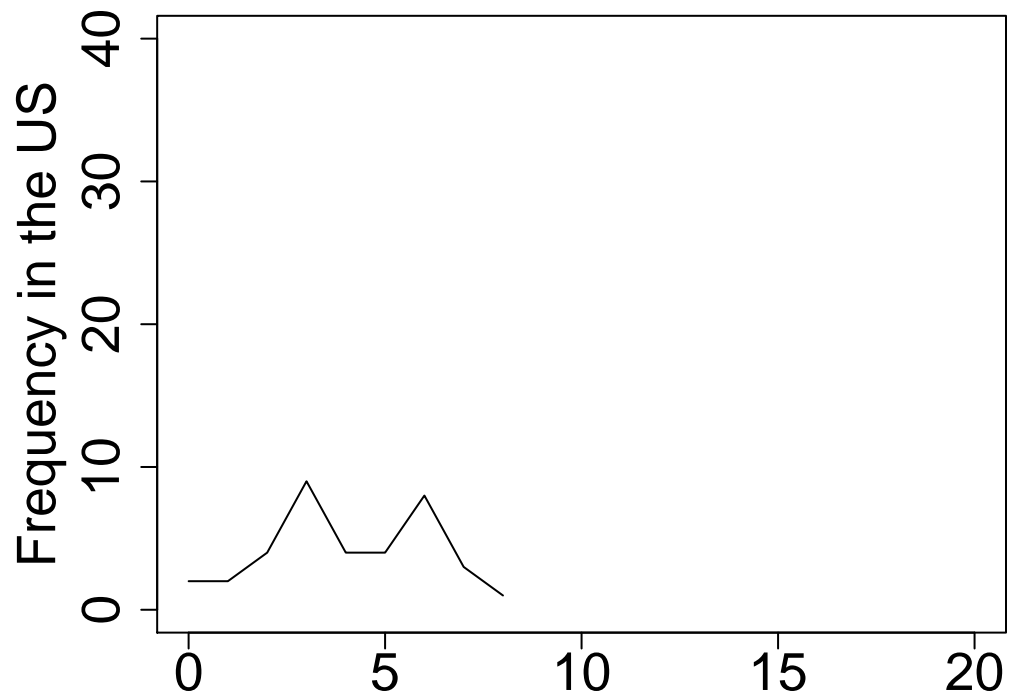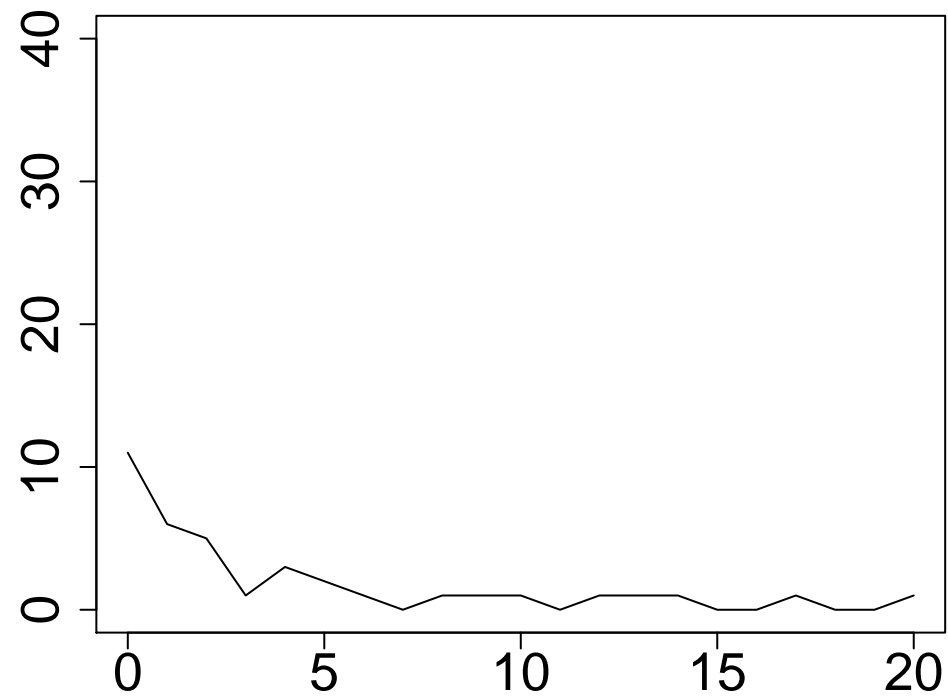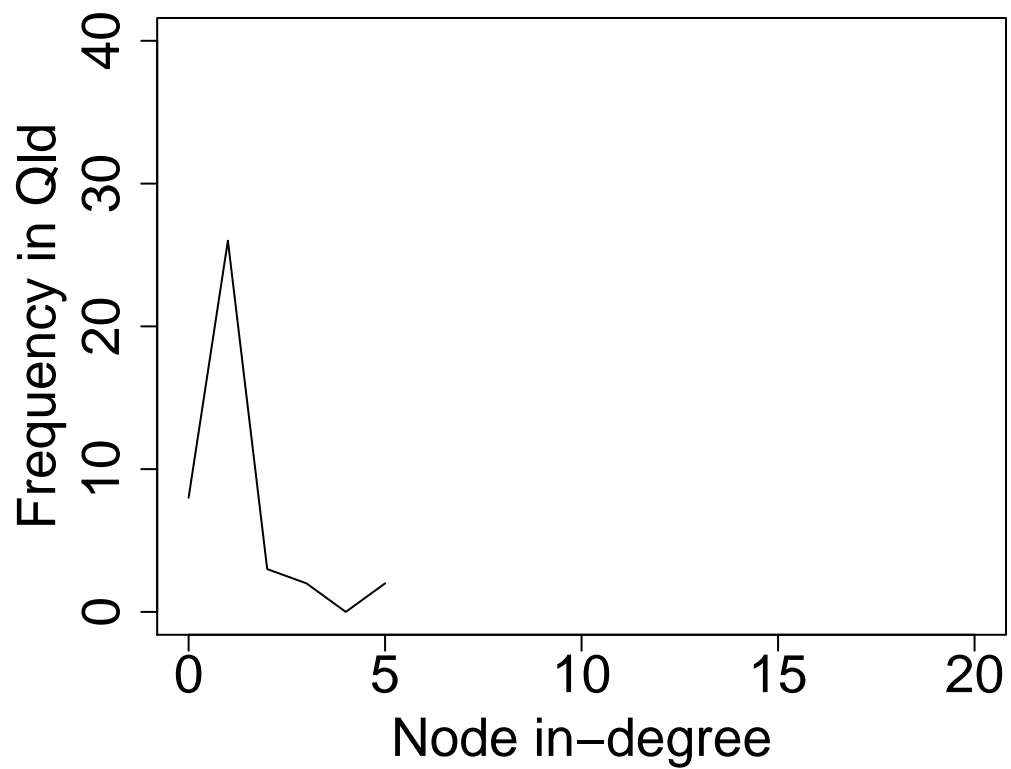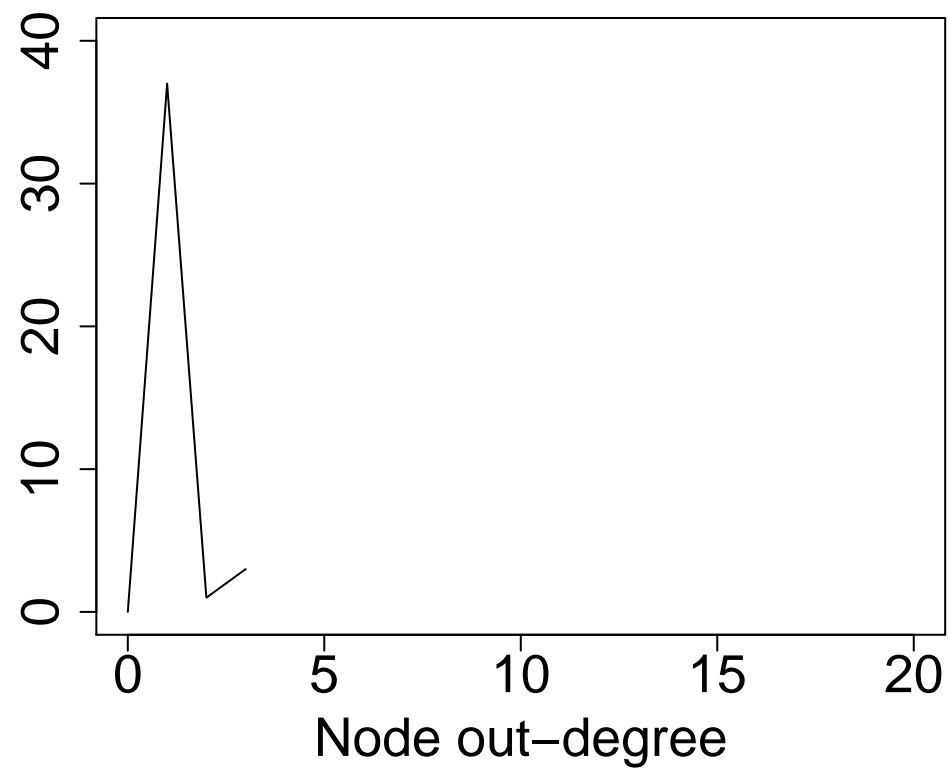

Supplement: SUPPLEMENTAL MATERIAL [file supp_biv122_suppl_data.zip › BioScience Fig. S2.pdf]

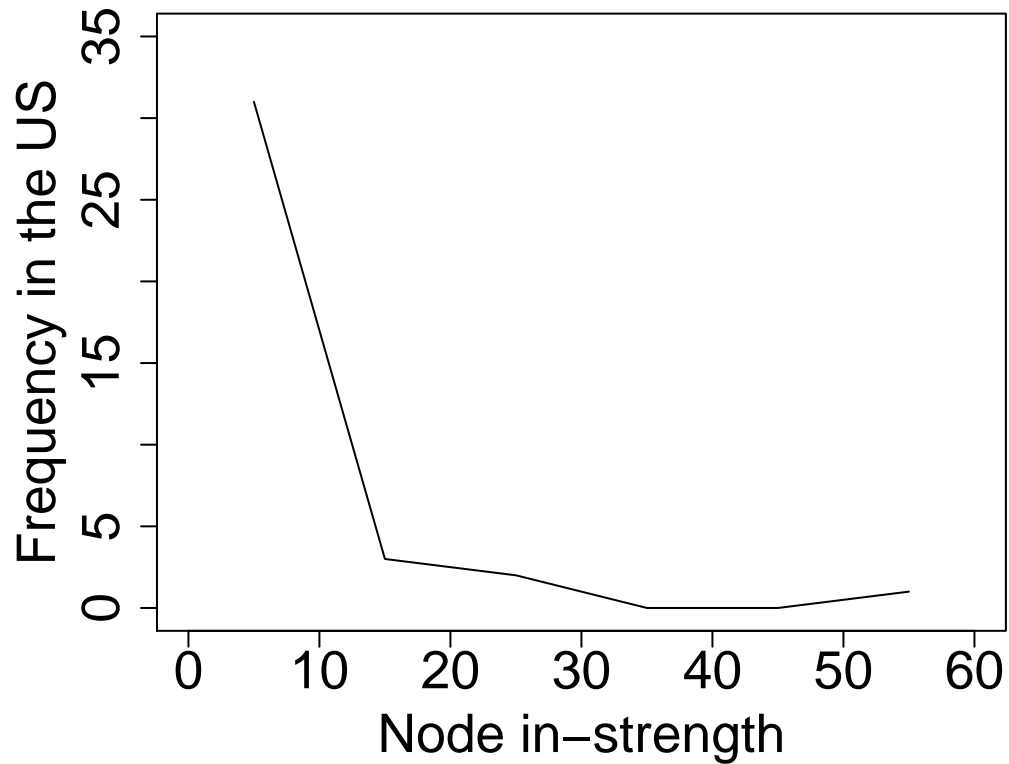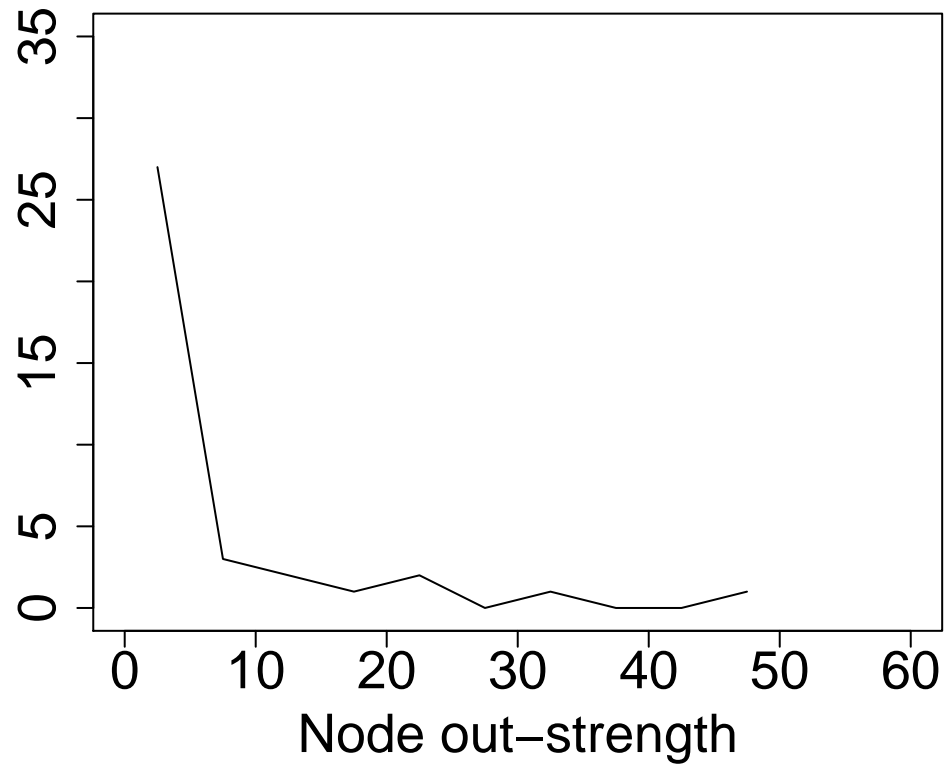

Supplement: SUPPLEMENTAL MATERIAL [file supp_biv122_suppl_data.zip › BioScience Fig. S3.pdf]

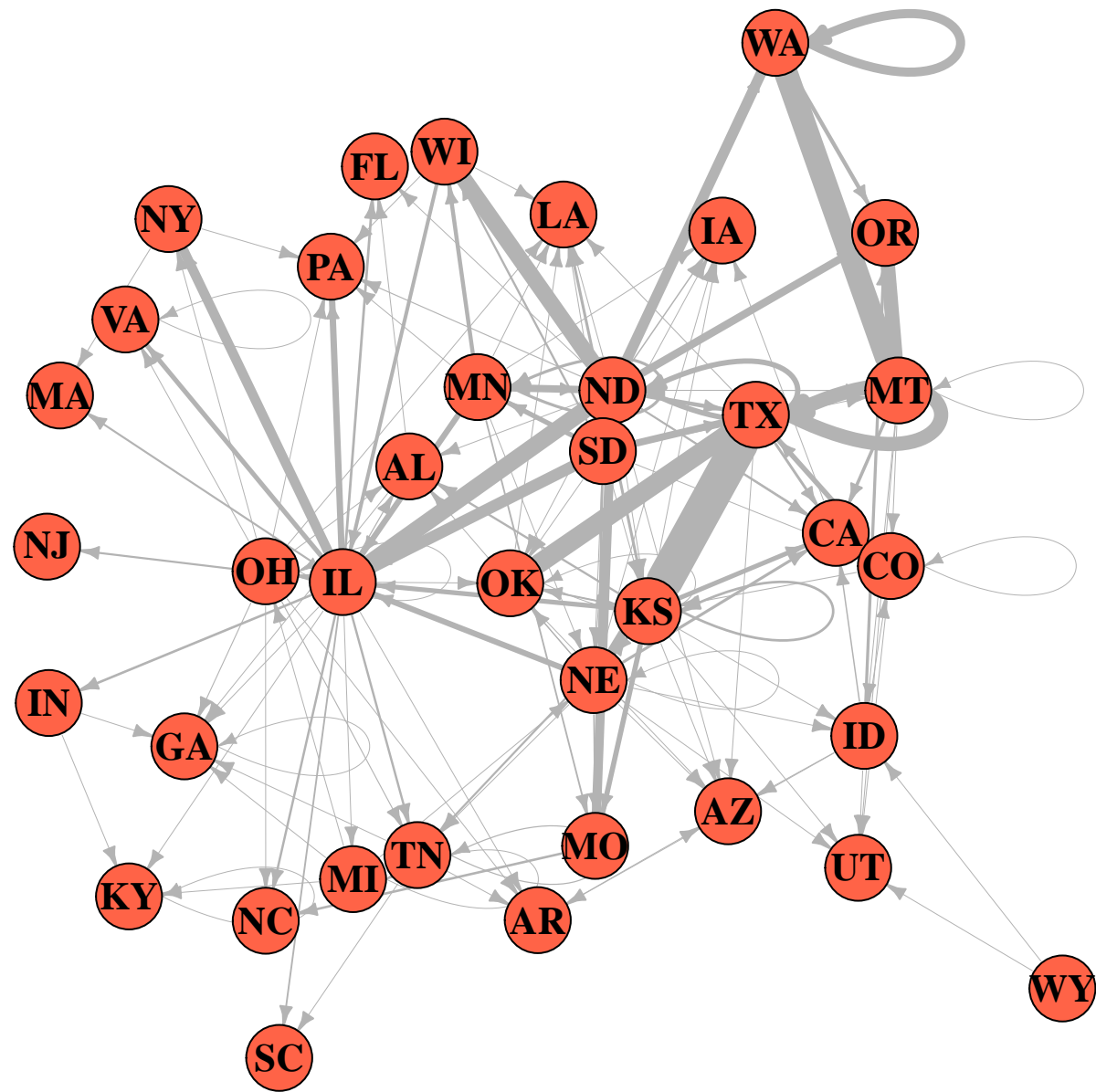

Supplement: SUPPLEMENTAL MATERIAL [file supp_biv122_suppl_data.zip › BioScience Fig. S4.pdf]
